# Enhanced sleep staging with artificial intelligence: a validation study of new software for sleep scoring

**DOI:** 10.3389/frai.2023.1278593

**Published:** 2023-12-05

**Authors:** Massimiliano Grassi, Silvia Daccò, Daniela Caldirola, Giampaolo Perna, Koen Schruers, Archie Defillo

**Affiliations:** ^1^Medibio Limited, Savage, MN, United States; ^2^Department of Biomedical Sciences, Humanitas University, Pieve Emanuele, Italy; ^3^Department of Clinical Neurosciences, Villa San Benedetto Menni Hospital, Hermanas Hospitalarias, Albese con Cassano, Italy; ^4^Humanitas San Pio X, Personalized Medicine Center for Anxiety and Panic Disorders, Milan, Italy; ^5^Department of Psychiatry and Neuropsychology, Faculty of Health, Medicine, and Life Sciences, Research Institute of Mental Health and Neuroscience, Maastricht University, Maastricht, Netherlands

**Keywords:** sleep staging, machine learning, deep learning, artificial intelligence, medical software

## Abstract

Manual sleep staging (MSS) using polysomnography is a time-consuming task, requires significant training, and can lead to significant variability among scorers. STAGER is a software program based on machine learning algorithms that has been developed by Medibio Limited (Savage, MN, USA) to perform automatic sleep staging using only EEG signals from polysomnography. This study aimed to extensively investigate its agreement with MSS performed during clinical practice and by three additional expert sleep technicians. Forty consecutive polysomnographic recordings of patients referred to three US sleep clinics for sleep evaluation were retrospectively collected and analyzed. Three experienced technicians independently staged the recording using the electroencephalography, electromyography, and electrooculography signals according to the American Academy of Sleep Medicine guidelines. The staging initially performed during clinical practice was also considered. Several agreement statistics between the automatic sleep staging (ASS) and MSS, among the different MSSs, and their differences were calculated. Bootstrap resampling was used to calculate 95% confidence intervals and the statistical significance of the differences. STAGER's ASS was most comparable with, or statistically significantly better than the MSS, except for a partial reduction in the positive percent agreement in the wake stage. These promising results indicate that STAGER software can perform ASS of inpatient polysomnographic recordings accurately in comparison with MSS.

## 1 Introduction

During sleep, our brain moves across a series of changes between different sleep stages characterized by specific brain and body activity patterns. Sleep staging refers to the process of mapping these transitions over a night of sleep (Danker-Hopfe et al., 2009). This is of great importance because sleep patterns, in combination with other features, provide the basis for diagnosing many sleep-related disorders and can add information crucial to characterizing several medical and psychiatric conditions associated with deteriorating sleep patterns.

Overnight polysomnography (PSG) is the primary diagnostic test used to evaluate patients with sleep problems. It consists of a set of signals, primarily from electroencephalogram, electrooculogram, electromyogram, and electrocardiogram data, recorded through sensors attached to different parts of the body.

The current gold standard for sleep staging is visual polysomnographic data review by certified sleep technicians following standardized rules of the American Academy of Sleep Medicine (AASM) (Ezzati et al., 2004) and based on the original criteria developed by Rechtshaffen and Kales (Kales et al., 1968). The standard classification defines five sleep stages following one another overnight: waking (wake), non-rapid eye movement (N1, N2, and N3), and rapid eye movement (REM).

Manual sleep staging (MSS) of sleep is a difficult, costly, and time-consuming process. In clinical practice, the classification of a full night (6-h recording) requires ~2–4 hours of visual scoring. Moreover, variability between independent scorers exists. The estimated agreement between scorers using AASM criteria ranges from 80 to 82% (Danker-Hopfe et al., 2009; Rosenberg and Van Hout, 2013; Younes et al., 2016). Indeed, the opportunity of automatizing this task would allow for not only substantial savings in time and costs but also a reduction in the between-scorer inconsistency (Wang et al., 2015; Sun et al., 2017). Moreover, it can help facilitate the transfer of sleep investigations from a clinical setting to the home environment, which will permit monitoring of the clinical course and therapy effects with long-term and frequent sleep assessments.

With the advancements in computer science, several supervised machine learning algorithms based on PSG-recorded signals have been proposed to perform automatic sleep staging (ASS), with hundreds of algorithms currently available in the scientific literature (Boostani et al., 2017; Faust et al., 2019). Some of these supervised machine learning algorithms rely on the calculations of some statistical parameters from PSG signals and use them as inputs. Other supervised machine learning algorithms based on deep learning (Goodfellow et al., 2016) allow for the direct use of raw PSG signals as inputs without the need for any prior feature extraction, thereby permitting sleep staging to be performed in a more end-to-end fashion. Some of the currently proposed machine learning algorithms have demonstrated excellent results, at the level of experienced technicians' agreement or greater (e.g., Nurma et al., 2016; Tsinalis et al., 2016; Zhang and Wu, 2017; Dimitriadis et al., 2018; Li et al., 2018; Patanaik et al., 2018; Stephansen et al., 2018; Feng et al., 2021; Fu et al., 2021; Perslev et al., 2021; Vallat and Walker, 2021; Bakker et al., 2023). While most of the proposed algorithms have been designed to use different signals as input, e.g., electroencephalography (EEG), electrooculography (EOG), and electromyography (EMG), some algorithms use EEG derivations solely as input (e.g., Peter-Derex et al., 2021; Sharma et al., 2021; Phan and Mikkelsen, 2022). However, most of them have remained mere prototypes and proofs of concept, and only a few have been validated and authorized by national regulatory bodies for their use in clinical practice (e.g., EnsoSleep by EnsoData, Madison, WI, USA).

We developed STAGER (Medibio Limited, Savage, MN, USA; CE Mark certification in 2020, Certificate Registration Number: 532495 MR6, Medical Device Directive of the European Union, Council Directive 93/42/EEC of 14 June 1993, OJ No L 169/1 of 1993-07-12), a software program based on a series of machine learning algorithms that performs ASS using PSG recordings. In general, despite the development details of the current ASS products have usually not been disclosed, they all seem to use information derived from multiple physiological signals, such as at EEG, EOG, and EMG, following the information normally taken into consideration by sleep technicians to accomplish this task. Instead, STAGER only relies on EEG signals to perform ASS. We aimed to validate the ASS performed by STAGER by investigating its level of agreement with both the staging performed during the clinical practice and an additional staging performed by three independent long-experienced sleep technicians in a sample of clinical PSG recordings randomly selected and collected retrospectively from multiple sleep clinics in Minnesota, US. Furthermore, this study introduces a novel analytical approach designed to directly compare the accuracy of STAGER's ASS with that of multiple technicians' MSS, thereby identifying which method yields greater accuracy in various sleep stages and sleep macrostructure indexes.

## 2 Materials and methods

### 2.1 Data collection

Forty EDF (European Data Format; Kemp et al., 1992) files were used to test the staging performance of the STAGER software, which contained the PSG recordings of subjects referred to sleep clinics for a sleep investigation, as prescribed by their physician. Of these, 20 files were from the Lakeland Health Services Sleep Center–Plymouth, 10 from Lakeland Health Services Sleep Center–St. Cloud, and 10 from Restore Sleep Center–Blaine, all of which are located in Minnesota, USA. The files were retrospectively collected among PSG studies consecutively recorded in each center in July 2019. The total number of forty EDF files was previously planned according to previous analysis related to the development of other automatic sleep STAGER software [e.g., MICHELE Sleep Scoring System by Younes Sleep Technologies, FDA 510(k) Number: K112102, https://www.accessdata.fda.gov/cdrh_docs/pdf11/K112102.pdf; SleepProfiler by Advanced Brain Monitoring, Inc., FDA 510(k) Number: K153412, https://www.accessdata.fda.gov/cdrh_docs/pdf15/K153412.pdf]. No data from these sleep centers have been used in the development of the ASS functionality of the STAGER software.

The associated clinical sleep staging reports and annotations made for clinical assessments, including lights-off and lights-on times, were also collected for each PSG. The only inclusion criteria were subject of age ≥18 years and completion of a full night's PSG study with over 6 hours of recording. No further inclusion or exclusion criteria were applied. The first forty consecutively recorded EDF files met the inclusion criteria and they were included in the analysis.

All EDF and associated files were de-identified by removing any personally identifiable information and assigned a progressive identification code before being collected. This study was approved by Western Institutional Review Board (Puyallup, WA, USA) (Approval no. 1230523) and conducted in accordance with the principles of the United States Food and Drug Administration Code of Federal Regulations Part 2, Good Clinical Practice (ICH., 1998), and the Declaration of Helsinki (World Medical Association., 2009).

### 2.2 Polysomnography

The EDF files contained the recordings of a full night's digital PSG study performed according to the AASM guidelines and acquired with the SomnoStar 10.2 sleep scoring system by Vyaire Medical, Inc. Among the several physiological channels recorded during the PSG, six EEG montages (F4A1, C4A1, O2A1, F3A2, C3A2, and O1A2; sampling rate, 200 Hz) were used by both STAGER software to perform ASS and the sleep technicians to perform MSS, both during the clinical practice and additional sleep staging by the three technicians described below (2.4); in addition, two electrooculographic (EOG) channels (E1 and E2; sampling rate, 200 Hz), and a single chin electromyographic (EMG) channel (sampling rate, 500 Hz) were also taken into account by the sleep technicians to perform MSS according to the AASM standardized rules.

### 2.3 STAGER software

The STAGER software is a medical software program for use with a Microsoft Windows operating system that analyzes previously recorded physiological signals from EDF files as per AASM guidelines. It initially performs sleep staging at every 30-s epoch of PSG recordings based on EEG signals. STAGER software uses input signals from the six EEG montages described above. The signals of these EEG channels are processed with a series of machine learning and deep learning algorithms and classified each 30-s epoch in one of five possible sleep stages: wake, N1, N2, N3, and REM. These algorithms were developed based on over a thousand EDF files from clinical PSG recordings. This represents more than 1 million 30-s epochs that were collected from multiple sleep centers, predominantly in North America, with one in Europe. All these PSG recordings pertain to patients who underwent PSG due to suspected primary or secondary sleep disorders. We used PSGs from patients aged 18 years or older in the training sample, with no data from pediatric patients included. Beyond this age criteria, no other exclusions were made regarding patient characteristics, ensuring a clinically representative sample for developing the STAGER's ASS functionality.

For each epoch, a spectral analysis of the EEG data is conducted, and the absolute and relative power values of EEG Alpha, Beta, Delta, Theta, and Sigma frequency bands are calculated, alongside other metrics and input. This information forms the basis the machine learning pipeline uses for automatic sleep staging. Initially, two machine learning algorithms independently perform an initial sleep staging for each 30-s epoch: one is a Convolutional Neural Network (CNN), and the other employs Gradient Boosting techniques. Then, two additional temporal-aware machine learning algorithms are applied: one is a deep learning model utilizing a recurrent neural network architecture, and the other is a further application of Gradient Boosting methods. Enhanced staging accuracy is achieved by contextualizing each epoch within the broader scope of the entire sleep recording. Finally, an ensemble method that integrates the outputs from the different algorithms is employed to determine the definitive sleep stage for each epoch.

STAGER includes a signal detector that identifies and rejects the signal of an epoch if a lack of signal is detected in at least half of the epoch (i.e., the absolute magnitude of the signal is <0.5 μV in at least 15 s). To solve the lack of signal quality, STAGER software can also perform ASS when at least either the montages C4A1, F4A1, and O2A1 or C3A1, F3A1, and O1A2 are available, although reduced accuracy is expected compared to the use of six EEG channels. Rather than filtering out EEG artifacts, such as those caused by eye or muscle movements, we exposed the machine learning algorithm to epochs containing these artifacts during training. This strategy enables the algorithm to utilize these artifacts as informative features for sleep staging, thereby enhancing the robustness and precision of sleep stage classifications.

The STAGER software requires the inclusion of the lights-off and lights-on times and performs staging of this part of the PSG recordings only.

For the validation phase, we included only EDF files not used in the development phase and collected from new sleep centers that did not have been involved in the development of the ASS functionality of the STAGER software; in addition, in the validation phase, no further changes were applied to the sleep staging algorithms to optimize the performance.

### 2.4 Technician-based MSS of the PSG recordings

For each EDF file, the original MSS of the PSG recording performed during the clinical practice was used for the validation. In all three sleep clinics, the standard clinical procedure implies that a technician initially performs the MSS on the night during the PSG study, and then the senior lead sleep technician reviews and finally approves the MSS on the following day, according to the AASM scoring guidelines.

In addition to the MSS conducted during the clinical routine, three experienced sleep technicians were enrolled in this study. These technicians have more than 15 years of experience in professionally annotating sleep studies and have an AASM certification. They all worked at the Lakeland Health Services Sleep Center in Plymouth. Each technician received the collected de-identified EDF files and was asked to independently stage each 30-s epoch of the PSG recordings assigning one of the five possible stages described above to each epoch, according to the AASM Manual for the Scoring of Sleep and Associated Events, Version 2.5 (Berry et al., 2018). All of them performed the staging using the Polyman software (Kemp and Roessen, 2007), Version 2.1.7273.58144 for Windows, which allows both epoch-by-epoch signal visualization and stage annotation. The three technicians performed their staging blindly to one another. In addition, since they may have previously contributed to the staging of some EDF files included in the validation phase during their clinical practice, the annotations previously reported, as well as, demographic variables such as subjects' age and sex, and other clinical information that could have contributed to creating an association with any previous clinical work were not shared with the technicians.

Finally, the STAGER software was used to perform ASS of the EDF files. As the program requires inputting the initial lights-off and final lights-on times before performing AS, these times were extracted from the annotation files to match those used in the clinical staging.

In both the MSS and ASS, a non-classified (U) stage was allowed to be assigned to an epoch if the signals were too corrupted for them to stage an epoch reliably, as indicated by the scoring AASM guidelines. The same criteria were valid for the previously performed routine clinical staging. In every analysis, U stages were removed, and only those epochs for which a stage was assigned by all scorings considered in that analysis were considered.

### 2.5 Statistical analysis

To validate the ASS performed by the STAGER software, we initially investigated its agreement with both the technicians' MSSs and the sleep staging performed during clinical practice for the PSG studies used in this study. First, we calculated the agreement of the ASS with the majority vote staging of the three technicians, overall and grouped by the five possible stages. Among the 30-s epochs, only those to which at least two of the three technicians assigned the same stage were used in the analyses and all others were discarded. A single majority vote stage was finally assigned to those epochs that were retained and this was used to calculate agreement with the ASS. This analytical strategy has been commonly used in validation studies of other medical software products that automate sleep staging (e.g., MICHELE Sleep Scoring System, SleepProfiler). A bootstrap resampling approach was applied (10,000 resamples, with resampling performed for entire EDF files), and the median of the bootstrap resampling distribution was used as a point estimate of the overall percent agreement (e.g., the overall accuracy; OPA), interrater reliability (as measured by Cohen's k), positive percent agreements (PPAs), negative percent agreements (NPAs), and positive predictive values (PPVs) with their respective 95% confidence intervals (CIs) based on the 2.5th and 97.5th percentiles of the distribution. Then, by using the same analytical strategy, we also calculated the agreement of the ASS with the clinical sleep staging, overall, and grouped by the five possible stages.

However, the strategy above does not allow performing a direct comparison between the accuracy of the software ASS and of the technicians' MSS, i.e., answering the question of whether the software is more accurate or less accurate than the technicians in performing sleep staging.

To do this, we investigated the difference between the average agreements of the MSSs performed by the three technicians and the average agreements of the MSSs among the technicians. This was performed by calculating the differences in the OPA and Cohen's k values of the ASS and MSSs, as well as the PPAs, NPAs, and PPVs grouped by the five possible stages. As the agreement of Technician 1 with Technician 2 is not equal to the agreement of Technician 2 with Technician 1, the average agreements of the MSSs among the technicians were based on an average of six values, two for each pair of technicians with both possible orders. Instead, only the average agreements of the ASS with the MSSs were calculated, not considering the agreements of the MSSs with the ASS. This strategy is closely in line with what Lovchinsky et al. (2019) recently proposed as an evaluation strategy for machine learning models when perfect human expert annotations are not possible. This approach also allows including all staged epochs in the analysis, circumventing the exclusion of non-consensus epochs inherent in the majority vote strategy.^1^ This adjustment prevents the potential overestimation of agreement between ASS and majority vote MSS, which could arise from the omission of the non-consensus epochs that are typically more challenging and ambiguous to score accurately. A bootstrap resampling approach was applied by performing a 10,000-resampling analysis of the EDF files. The median of the bootstrap resampling distribution was used as a point estimate of the differences in the PPAs, NPAs, and PPVs, and their respective 95% CIs based on the 2.5th and 97.5th percentiles of the distribution were calculated. A statistically significant difference was confirmed by both bounds of the CI being higher or lower than 0 (i.e., both having a positive or negative sign). Then, we also investigated the difference between the agreements of the ASS with the clinical staging and the average agreements of each individual technicians' MSS with the clinical staging. These analyses were also performed following the same bootstrap approach described for the previous analyses.

Finally, we evaluated the agreement between sleep macrostructure indices derived from the software ASS and those obtained from the technicians' MSSs as well as clinical staging. We also tested the difference in agreement between the ASS and the MSSs. Specifically, we examined total sleep time (TST), sleep latency (SL), REM sleep latency (REML), sleep efficiency (SE), wake after sleep onset (WASO), time in wake, time in N1 stage, time in N2 stage, time in N3 stage, and time in REM stage. In line with the previously described analytical methods, we computed the average intra-class correlation coefficients (ICCs) between the ASS and the three technicians' MSSs, as well as the average ICCs among the three technicians' MSSs alone. Similarly, we computed the ICCs between the ASS and the clinical staging, as well as the average ICCs between each technician's MSSs and the clinical staging. We also computed the differences between these ICCs to determine any significant disparity between the ICCs of the ASS and of the technicians' MSSs. To accomplish this, we employed the same bootstrap resampling method outlined above. For the ICC calculations, we used the absolute agreement definition for single measures under a fixed-rate scenario, corresponding to the ICC(A,1) model as described by McGraw (1996).

All analyses were performed in the Python programming language Version 3.7.3 (Python Software Foundation, 2019).

## 3 Results

**Figure 1:**
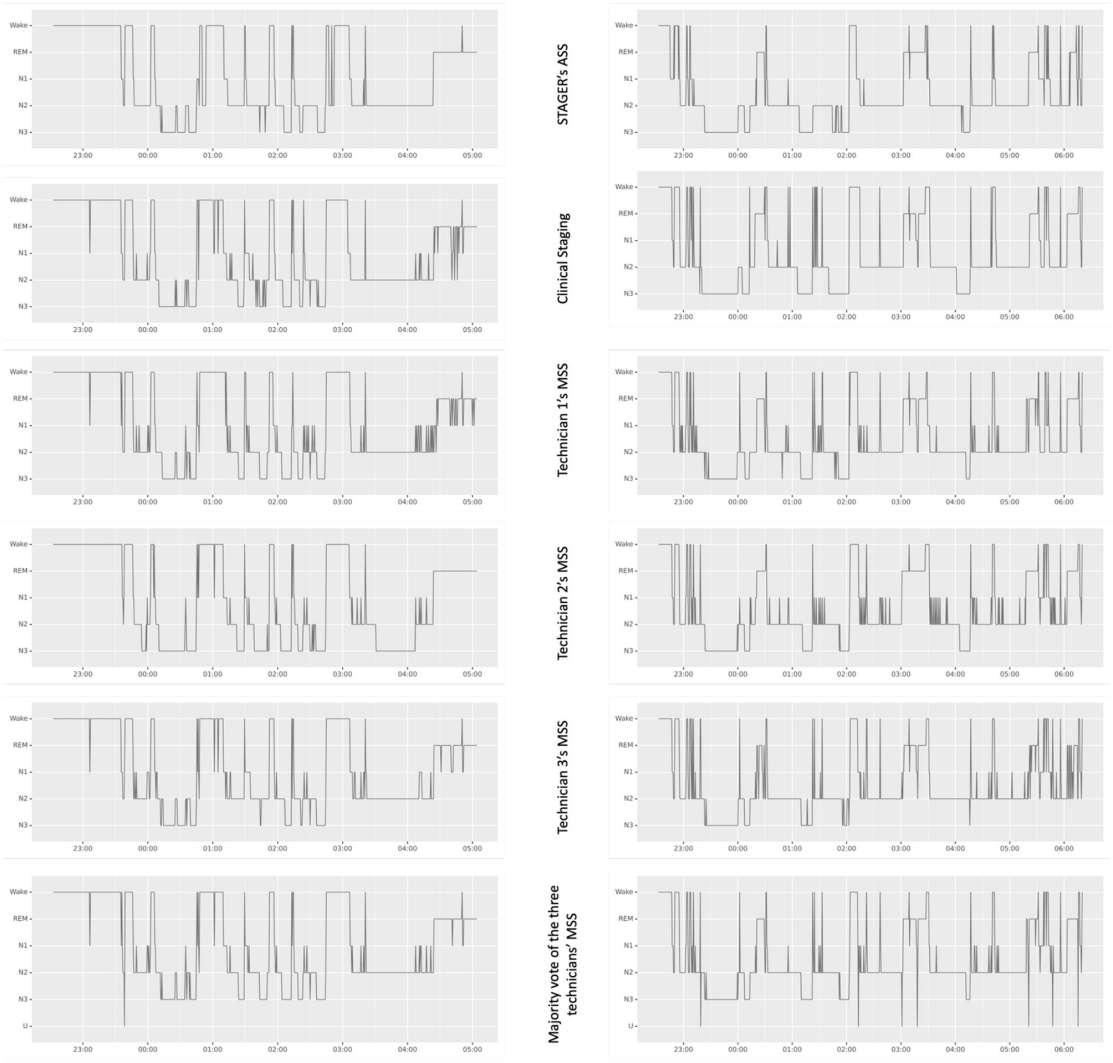
Hypnograms from STAGER's ASS, clinical staging, and technicians' MSSs of two polysomnography studies. This figure showcases a side-by-side comparison of hypnograms obtained from STAGER's ASS, clinical staging, and MSS performed by three expert technicians, including their majority vote staging for two PSG studies. These particular PSGs were selected based on STAGER's ASS achieving an OPA with the majority vote staging that is representative of the median performance across the study (OPA of the left PSG study: 87.99%; OPA of the right PSG study: 88.05%; median OPA of all studies: 88.02%). The label 'U' on the majority vote hypnogram signifies epochs where complete disagreement occurred among the three raters.

The age, sex, body mass index, and apnea–hypopnea index (AHI) of the recruited subjects as well as the lights-off time, lights-on time, and duration of the PSG studies are reported in Table 1. For all 40 subjects randomly sampled from the sleep centers, the clinical reason that prompted their need for PSG was suspected OSA. On the basis of the PSG data, 9 (22.5%) subjects did not have significant sleep apnea (AHI <5), 26 (65%) had mild sleep apnea (AHI ≥5 but <15), and 5 (12.5%) had moderate sleep apnea (AHI ≥15 but <30). None of the subjects had severe sleep apnea (AHI >30). In total, 18 males and 22 females were included in this study, and their ages and body mass indexes ranged from 26 to 84 years (mean = 49.58 years, standard deviation = 13.71) and from 23 to 49 kg/m2 (mean = 34.05 kg/m2, standard deviation = 6.54), respectively. It was possible to perform ASS in all epochs of all PSG recordings. The signals of the left EEG montages were detected as missing in a single epoch, and only the right montages were used to perform ASS in that epoch. No U stage was assigned by either STAGER or the three technicians during their MSS, whereas four U stages were found in the clinical staging. Figure 1 provides a visual comparison of the hypnograms for two representative PSG recordings, illustrating the correspondence between the different staging methods.

**Table 1:** Descriptive statistics of the subjects and polysomnography studies.

**Variable**	**Mean**	**Standard deviation**	**Minimum**	**Maximum**
Age	49.58	13.71	26	84
BMI	34.05	6.54	23	49
AHI	7.51	5.30	0.7	24
SpO_2_ <88%	1.34	2.18	0	10.2
Total arousal index	23.86	13.44	3.8	56.7
Lights-off	22:20:10	00:26:46	20:59:00	23:25:00
Lights-on	05:51:08	00:34:21	03:38:30	06:50:00
Duration	07:30:58	00:37:29	05:59:00	09:03:30
		***n***	**%**
Gender	m	18	45.00%
		f	22	55.00%

BMI, body mass index; AHI, apnea–hypopnea index.

### 3.1 Agreement of the software ASS with the majority vote of the MSSs performed by the three technicians

The resulting agreement of the ASS with the majority vote staging of the three technicians is reported in Table 2. A total of 36,030 epochs were staged by both STAGER and the three human raters. Of these, 493 (1.37%) were discarded from this part of the analyses as a majority vote could not be assigned to them. This rate is within the range of previously reported data of studies that used the majority vote approach (e.g., MICHELE validation study: 0.95%, https://www.accessdata.fda.gov/cdrh_docs/pdf11/K112102.pdf: Sleep Profiler validation study: 3.67%, https://www.accessdata.fda.gov/cdrh_docs/pdf15/K153412.pdf). A total of 8,574 epochs (23.80%) presented partial agreement between the three technicians (i.e., only two raters assigned the same stage to the epoch), and 26,963 epochs (74.83%) demonstrated full agreement between them.

**Table 2:** Agreement of STAGER's ASS with the majority vote staging of the three technicians.

								**PPA**			**NPA**			**PPV**		
		**WAKE**	**N1**	**N2**	**N3**	**REM**	**Total**	**Median**	**95% CI**		**Median**	**95% CI**		**Median**	**95% CI**	
Majority votes of the three technicians	Wake	6,478	902	190	10	37	7,617	85.11%	81.27%	88.32%	99.10%	98.74%	99.41%	96.26%	94.41%	97.66%
		N1	174	1,501	879	1	176	2,731	54.90%	50.29%	59.64%	95.15%	93.88%	96.22%	48.47%	44.59%	52.53%
		N2	59	636	12,888	559	221	14,363	89.78%	87.73%	91.56%	91.28%	89.91%	92.59%	87.49%	85.10%	89.58%
		N3	5	0	539	5,412	0	5,956	90.90%	87.25%	93.92%	98.11%	97.19%	98.78%	90.69%	86.47%	93.63%
		REM	14	61	239	0	4,556	4,870	93.69%	90.27%	96.16%	98.60%	98.10%	99.00%	91.36%	88.81%	93.63%
		Non-consensus	48	201	144	21	79	493									
		Total	6,778	3,301	14,879	6,003	5,069	36,030									

ASS indicates automatic staging; PPA, positive percent agreement; NPA, negative percent agreement; PPV, positive predictive value; 95% CI, 95% confidence interval by bootstrap resampling; REM, rapid eye movement.

Good OPA of the ASS with the majority vote staging of the three technicians (OPA = 86.78%, 95% CI = 85.36%−88.14%) and interrater reliability (Cohen's k = 0.8205, 95% CI = 0.8025–0.8382) were observed. The stage-specific PPAs also attained good results, with an N1 PPA of 54.90%, a wake PPA of 85.11%, an N2 PPA of 89.78%, an N3 PPA of 90.90%, and a REM PPA of 93.69%. Good results were also observed for stage-specific NPAs, with an N2 NPA of 91.28%, an N1 NPA of 95.15%, an N3 NPA of 98.11%, a wake NPA of 99.10%, and a REM NPA of 98.60%. Finally, good stage-specific PPVs were observed as well, with an N1 PPV of 48.4%, an N2 PPV of 87.49%, an N3 PPV of 90.69%, a REM PPV of 91.36%, and a wake PPV of 96.26%.

### 3.2 Agreement of the software ASS with the clinical staging

The resulting agreement of the ASS with the clinical staging is reported in Table 3. A total of 36,112 epochs did not present a U annotation in the clinical annotation file. All these epochs were also staged by STAGER and used to calculate the agreement scores. In addition, in these analyses, good OPA of 83.74% (95% CI = 82.53%−84.97%) and Cohen's k of 0.7796 (95% CI = 0.7642–0.7956) were observed, as were good stage-specific PPAs, with an N1 PPA of 50.98%, a wake PPA of 81.61%, an N2 PPA of 87.03%, an N3 PPA of 87.32%, and a REM PPA of 90.71%. Good results were observed for stage-specific NPAs as well, with an N2 NPA of 89.63%, an N1 NPA of 94.18%, an N3 NPA of 97.70%, a REM NPA of 98.18%, and a wake NPA of 98.50%. Good stage-specific PPVs were also observed, with an N1 PPV of 40.69%, an N2 PPV of 84.98%, an N3 PPV of 88.40%, a REM PPV of 89.34%, and a wake PPV of 93.78%.

**Table 3:** Agreement of STAGER's ASS with clinical staging.

								**PPA**			**NPA**			**PPV**		
		**Wake**	**N1**	**N2**	**N3**	**REM**	**Total**	**Median**	**95% CI**		**Median**	**95% CI**		**Median**	**95% CI**	
Clinical staging	WAKE	6,410	1,067	307	20	56	7,860	81.61%	76.58%	86.05%	98.50%	97.80%	99.04%	93.78%	90.72%	96.02%
		N1	316	1,343	808	4	157	2,628	50.98%	43.99%	58.47%	94.18%	92.79%	95.49%	40.69%	33.48%	48.51%
		N2	89	797	12,659	677	328	14,550	87.03%	84.87%	89.18%	89.63%	87.64%	91.44%	84.98%	81.61%	87.98%
		N3	4	0	770	5,302	0	6,076	87.32%	82.96%	91.23%	97.70%	96.66%	98.54%	88.40%	83.50%	92.58%
		REM	18	95	357	0	4528	4,998	90.71%	86.56%	94.06%	98.28%	97.63%	98.81%	89.43%	86.03%	92.32%
		Total	6,837	3,302	14,901	6,003	5069	36,112									

ASS indicates automatic staging; PPA, positive percent agreement; NPA, negative percent agreement; PPV, positive predictive value; 95% CI, 95% confidence interval by bootstrap resampling; REM, rapid eye movement.

### 3.3 Difference between the software ASS and the technicians' MSSs in their agreement with the average technicians' MSSs

The average agreements of the ASS with the MSSs performed by the three technicians, the average agreements of the MSSs among the technicians, and their differences are reported in Table 4. Both the OPA of the ASS with the MSSs and their interrater reliability were not statistically significantly different from those among the technicians' MSSs. The average PPA of the ASS with the MSSs was significantly better than that among the technicians' MSSs for N2 (median = 3.90%, 95% CI = 1.79% to 6.01%), statistically significantly worse for wake (median = −7.04%, 95% CI = −10.40% to −3.85%), and not significantly different for N1, N3, and REM. The average NPA of the ASS with the MSSs was statistically significantly better for wake (median = 1.16%, 95% CI = 0.64% to 1.67%) but not significantly different for N1, N2, N3, and REM. The average PPV of the ASS as compared with that of the MSSs was also statistically significantly better for wake (median = 3.55%, 95% CI = 1.90% to 5.212%) but not significantly different for N1, N2, N3, and REM.

**Table 4:** Differences between STAGER's ASS and technicians' MSSs.

		**Positive percent agreement**			**Negative percent agreement**			**Positive predictive value**		
		**Median**	**95% CI**		**Median**	**95% CI**		**Median**	**95% CI**	
Average agreement of STAGER with the three technicians	WAKE	83.02%	79.04%	86.33%	98.49%	97.92%	98.94%	93.61%	90.99%	95.67%
		N1	47.11%	42.45%	51.77%	94.55%	93.21%	95.67%	45.50%	42.19%	48.77%
		N2	87.34%	85.24%	89.20%	88.56%	87.16%	89.92%	83.07%	80.79%	85.16%
		N3	87.07%	83.60%	90.17%	97.75%	96.87%	98.42%	88.83%	84.95%	91.56%
		REM	91.60%	88.26%	94.18%	98.14%	97.58%	98.61%	88.55%	85.75%	91.08%
		Overall	83.30%	81.95%	84.67%						
		Cohen's K	0.7747	0.7577	0.7921						
Average agreement among the three technicians	WAKE	90.08%	87.23%	92.33%	97.33%	96.65%	97.89%	90.08%	87.23%	92.33%
		N1	47.86%	44.82%	50.86%	94.97%	94.27%	95.60%	47.86%	44.82%	50.86%
		N2	83.44%	81.57%	85.08%	89.25%	88.16%	90.30%	83.44%	81.57%	85.08%
		N3	85.89%	83.61%	87.85%	97.04%	96.44%	97.56%	85.89%	83.61%	87.85%
		REM	89.54%	87.40%	91.19%	98.31%	97.93%	98.62%	89.54%	87.40%	91.19%
		Overall	82.77%	81.31%	84.19%						
		Cohen's K	0.7686	0.7492	0.7873						
Difference	WAKE	−7.04%	−10.40%	−3.85%^*−^	1.16%	0.64%	1.67%^*+^	3.55%	1.90%	5.22%^*+^
		N1	−0.85%	−5.67%	4.63%	−0.43%	−1.33%	0.38%	−2.35%	−5.42%	0.58%
		N2	3.90%	1.79%	6.01%^*+^	−0.70%	−2.04%	0.68%	−0.38%	−1.95%	1.23%
		N3	1.25%	−1.87%	4.01%	0.72%	−0.23%	1.52%	2.90%	−0.82%	6.12%
		REM	2.12%	−0.67%	4.27%	−0.17%	−0.68%	0.31%	−0.96%	−3.57%	1.53%
		Overall	0.53%	−0.55%	1.60%						
		Cohen's K	0.0062	−0.0081	0.0204						

95% CI indicates 95% confidence interval by bootstrap resampling; REM, rapid eye movement; ^*+^, statistical significance with α = 0.05, with the average agreements of the automatic staging (AS) performed by STAGER with the manual stagings (MSSs) performed by the three technicians being higher than the average agreements of the MSSs among the three technicians; ^*^-, statistical significance with α = 0.05, with the average agreements of the ASS performed by STAGER with the MSSs performed by the three technicians being lower than the average agreements of the MSSs among the three technicians.

### 3.4 Difference between the software ASS and the technicians' MSSs in their agreement with the clinical staging

The average agreements of the ASS with the clinical staging, the average agreements of the technicians' MSSs with the clinical staging, and their differences are reported in Table 5. Both the OPA of the ASS with the MSSs performed by the three technicians and their interrater reliability were not statistically significantly different from those among the technicians' MSSs. Moreover, the average PPA of the ASS with the clinical staging was statistically significantly better than that of the technicians' MSSs with the clinical staging for N2 (median = 3.71%, 95% CI = 1.63% to 5.80%), statistically significantly worse for wake (median = −7.61%, 95% CI = −11.12% to −4.62%), and not significantly different for N1, N3, and REM. The average NPA of the ASS with the clinical staging was statistically significantly better for wake (median = 0.95%, 95% CI = 0.36% to 1.62%) but not significantly different for N1, N2, N3, and REM. The average PPV of the ASS as compared with that of the clinical staging was also statistically significantly better for wake (median = 2.80%, 95% CI = 0.74% to 4.92%) but not significantly different for N1, N2, N3, and REM.

**Table 5:** Differences across STAGER's AS, technicians' MSSs, and clinical staging.

		**Positive percent agreement**			**Negative percent agreement**			**Positive predictive value**		
		**Median**	**95% CI**		**Median**	**95% CI**		**Median**	**95% CI**	
Agreement of STAGER with the clinical staging	WAKE	81.73%	76.67%	86.16%	98.50%	97.80%	99.04%	93.72%	90.63%	95.99%
		N1	50.98%	43.99%	58.47%	94.17%	92.77%	95.48%	40.70%	33.49%	48.52%
		N2	87.03%	84.88%	89.19%	89.69%	87.68%	91.51%	85.11%	81.74%	88.09%
		N3	87.32%	82.96%	91.23%	97.69%	96.66%	98.54%	87.32%	82.96%	91.23%
		REM	90.71%	86.56%	94.06%	98.28%	97.62%	98.81%	89.45%	86.05%	92.32%
		Overall	83.77%	82.56%	85.00%						
		Cohen's K	0.7800	0.7646	0.7959						
Average agreement of the three technicians with the clinical staging	WAKE	89.42%	85.62%	92.56%	97.54%	96.69%	98.22%	90.89%	87.92%	93.29%
		N1	50.04%	45.76%	54.19%	94.45%	93.48%	95.35%	41.40%	34.31%	48.80%
		N2	83.32%	81.40%	85.11%	90.50%	88.82%	92.13%	85.68%	82.88%	88.33%
		N3	86.49%	82.88%	90.15%	97.06%	96.27%	97.75%	85.88%	81.97%	89.37%
		REM	90.64%	88.09%	92.60%	98.78%	98.40%	99.10%	92.42%	90.38%	94.22%
		Overall	83.72%	82.67%	84.77%						
		Cohen's K	0.7802	0.7659	0.7941						
Difference	WAKE	−7.61%	−11.20%	−4.62%^*−^	0.95%	0.36%	1.62%^*+^	2.80%	0.74%	4.92%^*+^
		N1	1.02%	−5.50%	7.99%	−0.28%	−1.21%	0.53%	−0.61%	−4.30%	2.88%
		N2	3.71%	1.63%	5.80%^*+^	−0.83%	−2.22%	0.51%	−0.59%	−2.24%	0.98%
		N3	0.81%	−2.97%	4.26%	0.62%	−0.20%	1.40%	2.52%	−0.74%	5.74%
		REM	0.12%	−2.84%	2.62%	−0.50%	−1.02%	−0.04%	−2.98%	−5.68%	−0.47%
		Overall	0.07%	−1.03%	1.05%						
		Cohen's K	0.0000	−0.0148	0.0133						

ASS indicates automatic staging; MSS, manual staging; 95% CI, 95% confidence interval by bootstrap resampling; REM, rapid eye movement; ^*+^, statistical significance with α = 0.05, with the agreement of the ASS performed by STAGER with the clinical staging being higher than the average agreements of the three technicians' MSSs with the clinical staging; ^*^-, statistical significance with α = 0.05, with the agreement of the ASS performed by STAGER with the clinical staging being lower than the average agreements of the three technicians' MSSs with the clinical staging.

### 3.5 Intra-class correlation of sleep macrostructure indices across ASS, MSS, and clinical staging

Table 6 summarizes the average ICCs for the comparison of the ASS with the technicians' MSSs, the average ICCs among the technicians' MSSs, and the differences between these ICCs for each sleep macrostructure index studied. With the exception of time in N1 stage, the ASS consistently exhibited good-to-excellent agreement (values <0.5, between 0.5 and 0.75, between 0.75 and 0.9, and >0.90 being indicative of poor, moderate, good, and excellent reliability, respectively, as per Koo and Li, 2016) with the technicians' MSSs (median ICC range: 0.845–0.9684), as did the agreements among the technicians' MSSs themselves (median ICC range: 0.8288–0.9676). Instead, both the average ICC of the time in N1 stage for the comparison of the ASS with the technicians' MSSs (median ICC: 0.6755) and the average ICC among the technicians' MSSs (median ICC: 0.7039) indicated a moderate level of agreement. For none of the indices was the difference in ICCs between the ASS and the technicians' MSSs statistically significant, with CI bounds not surpassing the threshold for significance (i.e., lower bound of the CI above 0, and upper bound of the CI below 0).

**Table 6:** Differences in the intra-class correlation coefficients of sleep macrostructure indices between STAGER's ASS and technicians' MSSs.

	**Average ICC between STAGER and the three technicians**			**Average ICC between the three technicians**			**Difference**		
	**Median**	**95% CI**		**Median**	**95% CI**		**Median**	**95% CI**	
Total sleep time	0.9684	0.9318	0.9845	0.9676	0.9154	0.9872	0.0016	−0.0146	0.0245
Sleep latency	0.8487	0.6662	0.9443	0.8731	0.7418	0.9485	−0.0226	−0.1386	0.0427
REM latency	0.9639	0.9053	0.9927	0.9541	0.8656	0.9992	0.0097	−0.013	0.0486
Sleep efficiency	0.9577	0.8955	0.9808	0.9568	0.8806	0.983	0.0016	−0.0234	0.0313
WASO	0.845	0.7497	0.9016	0.8288	0.7574	0.8981	0.0165	−0.0918	0.0909
Time in wake	0.9552	0.8885	0.9796	0.9526	0.8709	0.9813	0.0031	−0.022	0.0358
Time in N1	0.6755	0.5712	0.747	0.7039	0.5901	0.792	−0.0302	−0.123	0.0621
Time in N2	0.8705	0.7616	0.9298	0.8845	0.7888	0.9388	−0.0133	−0.061	0.0209
Time in N3	0.8521	0.738	0.9163	0.842	0.7405	0.9037	0.0116	−0.074	0.074
Time in REM	0.9489	0.9031	0.974	0.9669	0.9379	0.9826	−0.0171	−0.057	0.0084

ICC, intra-class correlation; 95% CI, 95% confidence interval by bootstrap resampling; REM, rapid eye movement; WASO, wake after sleep onset.

Similarly, Table 7 reports the ICCs for the ASS vs. clinical staging, the average ICC for the technicians' MSSs vs. clinical staging, and the ICC differences for each of the sleep macrostructure indices analyzed. In these analyses as well, with the exception of time in N1 stage, the ASS (median ICC range: 0.7649–0.9594) and the technicians' MSSs (median ICC range: 0.8134–0.9719) both demonstrated good-to-excellent agreement with clinical staging. Instead, both the time in N1 stage ICC for the comparison of the ASS with the clinical staging (median ICC: 0.4236) and the average ICC between the technicians' MSSs and the clinical staging (median ICC: 0.3867) indicated a poor level of agreement. Only the differences in the ICCs related to time in the REM stage reached statistical significance (median = −0.0361, 95% CI = −0.076 to −0.011), indicating a significantly lower ICC between STAGER's ASS and the clinical staging compared to the average ICC between the three technicians and the clinical staging (with both lower bounds of the CI below 0), despite both ICCs showed an excellent level of agreement (median ASS-clinical staging ICC: 0.9354; median average technicians' MSS-clinical staging ICC: 0.947). Moreover, although not significant and still indicative of a good level of agreement (median ICC: 0.7649), the ICC for sleep latency in the ASS vs. clinical staging was more than 0.1 lower than the average ICC for the technicians' MSSs vs. clinical staging (median ICC: 0.8738).

**Table 7:** Differences in the intra-class correlation coefficients of sleep macrostructure indices across STAGER's AS, technicians' MSSs, and clinical staging.

	**ICC between STAGER and the clinical staging**			**Average ICC between the three technicians and the clinical staging**			**Difference**		
	**Median**	**95% CI**		**Median**	**95% CI**		**Median**	**95% CI**	
Total sleep time	0.9594	0.9167	0.9809	0.9681	0.9333	0.9846	−0.0079	−0.0338	0.0092
Sleep latency	0.7649	0.4755	0.9272	0.8738	0.7608	0.9309	−0.1098	−0.3451	0.0283
REM latency	0.8126	0.4768	0.9931	0.8134	0.4806	0.9937	−0.0029	−0.0479	0.0415
Sleep efficiency	0.9472	0.8819	0.9761	0.9588	0.9091	0.9801	−0.0105	−0.0495	0.0112
WASO	0.795	0.5975	0.8976	0.8303	0.7298	0.89	−0.0344	−0.1845	0.0559
Time in wake	0.9434	0.8739	0.974	0.9546	0.9009	0.9776	−0.0102	−0.051	0.013
Time in N1	0.4236	0.0428	0.6537	0.3867	0.1095	0.5985	0.0283	−0.168	0.1917
Time in N2	0.8274	0.7226	0.8948	0.843	0.7604	0.8949	−0.0157	−0.084	0.043
Time in N3	0.8505	0.7019	0.9292	0.8312	0.7178	0.8942	0.0193	−0.058	0.0817
Time in REM	0.9354	0.883	0.9652	0.9719	0.947	0.9839	−0.0361	−0.076	−0.011^*−^

ICC, intra-class correlation; 95% CI, 95% confidence interval by bootstrap resampling; REM, rapid eye movement; WASO, wake after sleep onset. ^*−^, statistical significance with α = 0.05, with the ICC between the ASS performed by STAGER and the clinical staging being lower than the average ICC between the three technicians' and the clinical staging.

## 4 Discussion

The main aim of this study was to investigate the accuracy of the ASS that the STAGER software performs through a series of machine learning algorithms. This software is capable of performing ASS based on EEG signals only. To provide sound evidence of its staging performance when applied in the clinical setting, we used a sample of 40 clinical PSG recordings from three sleep clinics and calculated several agreement statistics of STAGER's ASS with both the manual staging performed during the clinical practice and an additional staging performed by experienced sleep technicians. All MSSs were performed using EEG, EOG, EMG signals, according to AAMS guidelines.

We decided to use PSG recordings of patients because (1) evidence has shown that sleep disorders may have disruptive effects on PSG signals and (2) the accuracies of both ASS and MSS in sleep patients are expected to be limited compared with what is achieved in healthy subjects (Boostani et al., 2017). Moreover, we performed an extensive investigation of the agreement of the ASS with two distinct MSSs combined with multiple analytical strategies, in terms of both direct AS–MSS agreement and the difference between agreements. Furthermore, given that one of the central issues in sleep staging is the lack of a perfect reference, with MSS by expert technicians being the current gold standard for sleep staging even if it has an expected interrater agreement of only 80–82% (Danker-Hopfe et al., 2009; Rosenberg and Van Hout, 2013; Younes et al., 2016), the use of two MSSs (the majority vote of three independent technicians and the staging performed in clinical practice) aimed to increase the strength of our results.

### 4.1 Agreement of the ASS with the MSSs

The ASS resulted in a particularly high agreement with the majority vote of the three independent raters (OPA = 86.78%), and a high, albeit partially reduced, agreement with the clinical staging (OPA = 83.74%). This outcome was expected, as the analysis with the clinical staging also included epochs on which the three raters had a full disagreement (i.e., those that were the most difficult to stage).

However, these results do not provide direct evidence of the accuracy of ASS in comparison with that of MSS. For this reason, we performed additional analytical strategies beyond the commonly used majority vote that aimed to allow for a more direct comparison between ASS and MSS. The ASS performed by the STAGER software was either comparable with or better than the MSSs performed by three technicians in all agreement statistics except for the PPA of the wake stage, which was partially reduced. Specifically, the ASS of N1, N3, and REM showed comparable results with those of the MSSs of the same stages. By contrast, for the N2 stage, the ASS resulted in a significantly better PPA and comparable NPA and PPV. Finally, taking into consideration the wake stage, its NPA and PPV under the ASS were significantly better than those under the MSSs, whereas its PPA was the sole agreement statistic for which the ASS was not comparable with or better, but statistically significantly worse, than the MSSs.

The use of EEG signals only as input information for the ASS system may be, at least in part, the reason for the reduced performance of the wake stage in PPA, especially considering that most of the non-agreements in the staging of wake were toward the N1 stage. The similarity in the EEG signal patterns of the wake and N1 stages, whose transition is characterized by a progressive shift from alpha to theta waves, may make these two stages difficult to distinguish, especially with a lack of additional eye and muscle movement information. Future versions of the staging system might include additional signals, such as EOG and EMG signals, to improve the PPA of the wake stage while maintaining the excellent staging performance accomplished in the other stages.

A notable observation from our study is the lower agreement rate for the N1 stage, which is consistent with what is generally observed in manual sleep stage scoring by trained technicians (Danker-Hopfe et al., 2009). For example, the average positive percent agreement for the N1 stage among the three technicians was 47.86%, which is not statistically different from the 47.11% average positive percent agreement observed between the ASS model and the technicians. This underscores the challenge in N1 stage classification that is commonly encountered, both in ASS by automated systems and in MSS by technicians.

Complementing the analysis on categorical agreement, the assessment of sleep macrostructure indices through ICCs reinforced the reliability of STAGER's ASS. The consistently good-to-excellent ICC values between the ASS and both the technicians' MSSs and the clinical staging (as reported in Tables 6, 7) underscore the precision of the ASS in quantifying sleep parameters. In line with the limited agreement observed for the N1 stage in the categorical agreement analysis, only the ICCs related to the time in N1 stage resulted of poor-to-moderate in magnitude. Moreover, the predominance of non-statistically significant differences in the ICCs when comparing the ASS with the technicians' MSSs indicates that the software's accuracy in determining sleep macrostructure indices is comparable with that of the technicians. Even for the time in REM and the sleep latency ICCs, for which the results may suggest a reduced agreement of STAGER's ASS with the clinical staging with respect to the technicians's MSS agreement with the clinical staging, STAGER's ASS ICCs were respectively excellent and good in magnitude (Koo and Li, 2016). Overall, these results align with those observed in the analysis of categorical agreements for each sleep stage.

Various machine learning-based algorithms that conduct ASS exclusively using EEG have been presented in scientific papers (Phan and Mikkelsen, 2022). However, directly comparing the performance of STAGER with these algorithms is challenging due to the distinct datasets and methodologies employed in our study. Some of these algorithms also demonstrated a high level of agreement with human scorers testing on truly independent data, [e.g., Vallat and Walker, 2021, in which the algorithms proposed by Stephansen et al. (2018) and Perslev et al. (2021) are also further tested]. This implies the use of PSG studies collected in clinical settings different from those used in any phase of their algorithm's development. Nevertheless, what distinguishes our study is its focus on investigating the performance of an ASS tool across different types of MMS and novel analytical strategies. This highlights the present strengths and limitations of ASS performed by STAGER, paving the way for more informed adoption, and pinpoints areas for improvement in subsequent versions.

### 4.2 Limitations

We used a random sampling approach to select the PSG recordings included in the study. However, all the selected PSG recordings referred to subjects who underwent PSG for suspected OSA. None of these subjects was found to have severe OSA (AHI >30), other sleep disorders (e.g., restless leg syndrome and REM sleep behavior disorder), or other disorders potentially affecting the performance of systems that base ASS on EEG signals (e.g., epilepsy). Future investigations should focus on testing the performance of STAGER's ASS in patients with these attributes as well. In addition, future studies are needed on the ASS model performance in healthy control subjects who have not been referred to a sleep clinic for a suspected sleep disorder. Moreover, even if we used data from three sleep centers and three independent raters, it is worth highlighting that the three sleep centers were managed by the same organization and that the three independent raters all worked for some of these sleep centers. Evidence has shown that technicians working in the same clinic tend to agree more than technicians working for different institutions do (Norman et al., 2000). Indeed, in our analysis, we observed a between-technician agreement for the N3 sleep stage that exceeded the levels reported in certain studies (e.g., 67.4% in Rosenberg and Van Hout, 2013). However, this was not universally the case, as other studies have also reported high N3 agreement between raters from different institutions (e.g., Cesari et al., 2021). Moreover, the 74.83% rate of full consensus among the three raters observed in our study is larger than that observed in other studies (e.g., 58.6% in Bakker et al., 2023). However, this potentially increased interrater agreement may have also resulted in a reduced AS–MSS agreement and not necessarily in favor of our results.

Finally, to avoid the risk that raters may associate PSG recordings, we did not provide them with any additional information beyond the PSG signals, including lights-off and lights-on times. Because of this, raters missed ending the staging of the PSG recordings exactly at the same epoch; therefore, some epochs at the end of the recordings were not considered in the analyses. However, the number of missed epochs was limited (*n* = 86, 0.24%), and there is no reason to expect an a priori difference in the AS–MSS agreement in these late epochs compared with the preceding ones that we were able to consider.

### 4.3 Conclusions and future directions

The burden associated with the MSS process makes the availability of good ASS applications particularly relevant in both research and clinical practice. MSS requires significant training and experience before sleep technicians can achieve good staging ability. Moreover, MSS can take even more than 2 hours per PSG recording and is a repetitive task that can be affected by tiredness, distraction, and time pressure. Because of this high demand for manual work, the costs associated with PSG are significant and the reliability of staging cannot always be guaranteed.

By contrast, automatizing sleep staging can lead to several advantages. First, ASS applications allow for a significant reduction of the staging time. For example, the current version of the STAGER software, which runs on Windows 10, takes 5 minutes on average to stage an entire PSG recording and has been proven to be able to complete ASS of a full PSG recording in <2 minutes on a professional-grade laptop and other operating systems. If we consider that, beyond speed, ASS software may also tirelessly process PSG recordings without interruption, the widespread use of ASS applications may allow for significant cost reductions in sleep investigations. First, however, sound evidence of ASS's safety and accuracy should be established and authorization from national regulatory bodies, as expected for any other medical device and software, should be obtained for it to be safely implemented in clinical practice. All other ASS applications currently available are intended to be used as a supporting tool for clinicians—that is, not to perform fully autonomous sleep staging but instead to provide an initial ASS that has to be later manually reviewed and approved by trained technicians. The more ASS systems are proven to achieve a performance comparable with that by human raters, especially in epochs with unusual or unexpected signal patterns, the fewer human revisions will be needed and the more joint machine–human work will be expected to lead to improved scoring, especially considering that ASS systems and human raters may be prone to errors in different scenarios.

Another, and usually less highlighted, advantage of ASS systems is that they can perform self-consistent scoring—that is, they always lead to identical results while processing the same PSG recording. By contrast, MSS is not self-consistent: different scorers are expected to show disagreement in the scoring, and even the same scorer may provide a different scoring to the same PSG recording if asked to repeat the scoring at different times. This affects not only clinical results but also research studies, thereby introducing variability both within and between study results. Therefore, using the same ASS system may help make the results among different PSG recordings, sleep clinics, and research studies more consistent and thus more directly comparable with one another.

In conclusion, this study has performed an extensive evaluation of the STAGER software's ASS system. Its ASS was, for the most part, comparable with or even better than the MSSs performed by human raters, with the sole exception of a partial reduction in the PPA for the wake stage. These promising results indicate that the use of STAGER may help reduce the burden usually associated with full MSS of an inpatient PSG recording. Further studies will be performed to investigate specific moderators of the system's accuracy. Moreover, the next version of the STAGER ASS system will focus in particular on improving the PPA of the wake stage and on achieving ASS with a different set of signals that may more easily be collected with devices used in the home environment.

## Data availability statement

The datasets presented in this article are not readily available because the dataset is Medibio Limited property. Requests to access the datasets should be directed to massimiliano.grassi@medibio.com.

## Ethics statement

The studies involving humans were approved by the United States Food and Drug Administration Code of Federal Regulations Part 2, Good Clinical Practice (ICH., 1998), and the Declaration of Helsinki (World Medical Association., 2009). The studies were conducted in accordance with the local legislation and institutional requirements. The participants provided their written informed consent to participate in this study.

## Author contributions

MG: Data curation, Investigation, Conceptualization, Formal analysis, Methodology, Software, Validation, Writing—original draft. SD: Investigation, Supervision, Writing—review & editing. DC: Investigation, Supervision, Writing—review & editing. GP: Supervision, Writing—review & editing. KS: Supervision, Writing—review & editing. AD: Resources, Supervision, Writing—review & editing, Conceptualization, Funding acquisition, Investigation, Project administration.

## Footnotes

^1^Recently, the application of the hypnodensity approach has extended to MSS, providing a representation of the distribution of manual sleep stages as probabilistic outputs rather than discrete classifications, when sleep staging performed by multiple scorers is available (Anderer et al., 2023). This method depicts the level of agreement and uncertainty among sleep technicians when assigning sleep stages, thereby capturing the inherent variability and subjectivity of manual scoring. However, considering the unique context of our study, we opted to not incorporate the hypnodensity approach into our analysis. The primary reason for this decision was the limited number of independent scorings available, which could result in an imprecise estimation of MSS probabilities. In studies where the hypnodensity approach has been successfully applied (Stephansen et al., 2018; Bakker et al., 2023; Fiorillo et al., 2023), a minimum of five scorers was utilized to generate reliable MSS probabilities. Our study, in contrast, included four MSSs, with only three being fully independent and based on the same scoring procedure. Given this, we considered the hypnodensity approach was not suitable for the analysis of the current study.
